# Analytical Validation of Multimodal AI Test Predicting Breast Cancer Recurrence Risk (Ataraxis Breast RISK)

**DOI:** 10.3390/diagnostics16071023

**Published:** 2026-03-29

**Authors:** Marc Dantone, Martin Lacsamana, Ken G. Zeng, Paraic A. Kenny, Krzysztof J. Geras, Jan Witowski

**Affiliations:** 1 Ataraxis AI, New York, NY 10016, USA; 2 Gundersen Health System, La Crosse, WI 54601, USA

**Keywords:** artificial intelligence, digital pathology, breast cancer, analytical validation

## Abstract

**Background/Objectives:** Breast cancer recurrence risk stratification has relied on gene expression tests that are limited by long turnaround times and consumption of valuable tissue. Artificial intelligence (AI) utilizing digital pathology images elucidates novel morphological biomarkers with strong prognostic associations, but the use of such AI models requires a modified analytical validation approach. Here, we report analytical validation of a novel breast cancer prognostic test. **Methods:** Ataraxis Breast RISK (ATX) uses a survival analysis model based upon features from a pan-cancer foundation model. This model extracts morphological features (biomarkers) from H&E-stained slides. These features are combined with clinical variables, and the test outputs a calibrated recurrence risk score. We validated ATX across five axes: intra-operator repeatability, inter-operator reproducibility, limit of blank, limit of detection and inter-laboratory reproducibility. Additionally, we assessed robustness to clinicopathologic data perturbations and conducted a clinical validation bridging study. Experiments were performed in CLIA-certified laboratories. **Results:** Intra-operator repeatability yielded an intraclass correlation coefficient (ICC) of 0.99 with 100% risk category agreement. Inter-operator reproducibility was concordant (ICC 0.99, 100% agreement). Inter-laboratory reproducibility across multiple scanners showed an ICC of 0.97 with 94.7% agreement. Under simulated clinicopathologic data perturbation, ATX maintained an average C-index of 0.62 with 90.0% agreement. The bridging study confirmed that the performance of the CLIA version was comparable to the prior clinical validation version (C-index 0.63 vs. 0.62). **Conclusions:** ATX met all predefined analytical acceptance criteria. These results support the analytical readiness of ATX use in clinical testing.

## 1. Introduction

Over the last two decades, patient risk stratification in breast cancer has been based primarily on clinicopathological features, such as stage or molecular subtype, in addition to gene expression profiling tests. Although these assays helped evaluate the risk of cancer recurrence and survival since their development in early 2000s, they suffer from workflow limitations, such as turnaround time ranging from 2 to 6 weeks and exhausting valuable tissue. However, since these assays use well-known and standardized laboratory techniques, they have been thoroughly validated with regard to their clinical performance and analytical reliability.

Artificial intelligence (AI) applied to digital pathology enabled discovery of new morphological biomarkers that are strongly associated with patient outcomes [1,2]. The first tests incorporating AI and digital pathology have been developed and reported to have promising results in clinical and analytical validation studies [3,4]. However, in the last several years, the emergence of self-supervised learning and foundation models changed the approach to the development of novel diagnostic tools, as they do not rely on predefined biomarkers [5,6,7,8,9]. This change requires a modified approach to analytical validation (AV) procedures.

In this study, we perform the first reported AV of a breast cancer laboratory-developed test (LDT) built based on a survival analysis model utilizing self-supervised learning features. The evaluated test, Ataraxis Breast RISK (ATX), integrates morphological features extracted from diagnostic H&E-stained slides with clinical features to produce a recurrence risk score as well as a “high” or “low risk” result based on a predefined threshold of 0.1. ATX was first developed on a cohort of 4659 patients and demonstrated consistent performance across five independent external cohorts (N = 3502), achieving a pooled C-index of 0.71 (0.68–0.75) [5]. Notably, the test shows promise for key clinical subgroups, including premenopausal patients [10] and clinically high-risk patients eligible for adjuvant CDK4/6 inhibitors [11]. This study aims to assess the reproducibility and robustness of the test under the defined study conditions.

## 2. Materials and Methods

### 2.1. Ataraxis Breast RISK Overview

ATX first extracts morphological features using a pre-trained pan-cancer foundation model for digital pathology. We have previously developed a foundation model, Kestrel, which was shown to be highly performant across a variety of pan-cancer tasks. Kestrel is a vision transformer-based [12] AI foundation model trained with a DINO-style [13] objective. The foundation model generates patch-level embeddings, which are aggregated into a slide-level representation using gated attention. The development of Kestrel, including model architecture, training data size and training objectives were previously described in detail in other studies [14].

**Figure 1:**
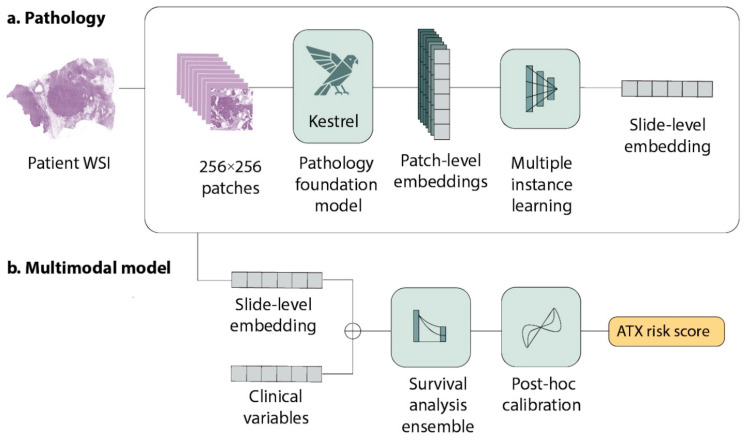
Components of Ataraxis Breast RISK (ATX). (**a**) For the pathology component, whole-slide images are divided into 256 × 256 patches. Patches with less than 30% foreground, as determined by Otsu’s method [15], were excluded. Patch-level embeddings are generated using Kestrel and subsequently aggregated into a slide-level representation via multiple instance learning. (**b**) For the multimodal component, the slide-level representation is combined with clinical variables and used as input to an ensemble of deep learning-based Cox models. The predictions from the ensemble are calibrated to estimate 5-year recurrence probabilities using spline-based calibration.

Then, ATX performs multimodal aggregation and combines digital pathology-extracted features with a set of clinical features: age, tumor size, nodal status, ER/PR/HER2 status, and histologic subtype. Finally, ATX uses an ensemble of Cox proportional hazards neural networks to produce a continuous score between 0 and 1 indicating a predicted probability of recurrence at a selected clinically relevant time horizon. This raw output is then calibrated such that the score represents a calibrated probability. In this analytical validation study ATX, the output time horizon is selected to be 5 years. All components of ATX (see Figure 1) were locked prior to analytical validation. ATX is currently performed as a laboratory-developed test (LDT) in a single CLIA-certified laboratory in Orlando, Florida.

### 2.2. Study Design and Procedures

The analytical validation study included several components: intra-operator repeatability, inter-operator reproducibility, limit of blank (LOB) and limit of detection (LOD) controls, inter-laboratory (inter-scanner) reproducibility, robustness to clinicopathologic data perturbations, and clinical validation bridging study. A plan for these analyses was pre-specified, with intra-operator repeatability and inter-operator reproducibility considered as primary analyses and the remaining as secondary. Positive readout of primary analyses was required for the analytical validation study to be considered successful.

We identified 175 archival cases of invasive breast cancer with available H&E-stained slides (one slide per patient) with matching clinical information. The cases represented a range of clinical risk profiles. This cohort was previously used as an external test set in a clinical validation study confirming ATX’s prognostic performance [5]. These cases were not used to modify ATX before or after clinical validation study. In addition to 175 patient slides, the study included control slides for LOB and LOD testing, which were processed to confirm failure detection due to absence of tumor tissue. The LOB slide consists of a blank glass slide. The LOD slide consists of a slide that does not contain tumor cells consistent with the originally diagnosed breast cancer subtype. The two slides were passed through the CLIA workflow and software as controls and the LOB/LOD checks were evaluated qualitatively (pass/fail). The analysis was considered successful if the process flagged the control as invalid by the board-certified pathologist in the laboratory prior to score generation.

Out of 175 archival cases, we excluded 9 patients with missing information about cancer recurrence and 6 patients who did not pass pre-specified quality control checks (sufficient tumor content or consistency with the provided diagnosis). Ultimately, the study included 160 cases with 19 observed recurrence events. Given that primary analyses require repeated processing and scanning, we further selected a subset of 30 representative cases for inter- and intra-operator experiments. We used the full set of 160 cases for all the secondary analyses. Population characteristics of the 160-patient cohort can be found in Table 1.

All experiments were performed in Ataraxis AI CLIA-certified laboratory. Additionally, for inter-laboratory (inter-scanner) reproducibility analysis, cases were processed in the CAP-accredited Gundersen Cancer Biobank laboratory. All cases were accessioned and scanned following pre-defined standard operating procedures (SOPs) in both laboratories. All cases underwent quality review by a board-certified pathologist prior to results generation. Slides were scanned at 40x using the primary whole-slide scanners, Grundium Ocus 40 in Ataraxis AI CLIA laboratory and Motic EasyScan Pro 24 in Gundersen Biobank.

### 2.3. Repeatability and Reproducibility

Intra-operator repeatability reflects the consistency of results when the same operator repeats the assessment over three days. On each day, the same set of 30 cases were processed, generating 90 ATX scores in total. Inter-operator reproducibility reflects the consistency of results when two operators process the same set of 30 cases, generating 60 ATX scores in total.

### 2.4. Clinical Validation Bridging Study

In the clinical validation study, the development version of ATX was found to be prognostic in early-stage invasive breast cancer [5]. To establish that the version of ATX offered in the CLIA laboratory setting has clinical performance comparable to that previously reported for ATX, we performed a bridging study. For this experiment, clinical performance of ATX was measured using scores for all 160 cases included in this study and compared to performance using scores from the previously reported development version of ATX. We measure prognostic performance of both versions of ATX with Harrell’s C-index with 95% confidence intervals.

### 2.5. Robustness to Realistic Data Variability

To evaluate robustness to realistic upstream clinicopathologic variability, as well as to account for discrepant or incorrect clinical inputs that could occur in real world testing, we performed a sensitivity analysis informed by published discordance rates for ER, PR, HER2, and histologic classification (5–15%) [16,17,18,19]. A uniform 10% perturbation was selected as a conservative approximation within reported real-world ranges. This procedure was repeated across 100 random draws to generate confidence intervals for model performance under plausible clinical variability. All metrics were computed separately for each of the 100 resampled datasets, and confidence intervals were derived from the distribution of these resampled estimates.

For ordinal variables (pathologic T stage and N stage), each non-missing value was assigned a 10% probability of perturbation. When perturbation occurred, the stage was randomly shifted by one adjacent category (either upward or downward) within predefined ordinal hierarchies. T stage transitions were restricted to the ordered sequence T0, T1mi/a/b, T1c, T2, T3, and T4. N stage transitions were restricted to the ordered sequence N0, N1, N2, and N3. Boundary constraints were enforced to prevent transitions beyond the lowest or highest category. Binary variables indicating subtypes, IDC and ILC, were independently flipped with a probability of 10%. For binary biomarker variables, positive values were converted to negative with a 10% probability, while negative values remained unchanged. Similarly, for HER2 status, positive or equivocal values were converted to HER2-negative values with a 10% probability. These one-directional perturbations simulate conservative misclassification scenarios. Continuous age values were perturbed by adding zero-mean Gaussian noise with a standard deviation of 1 year. After re-sampling, age values ranged from 27.0 to 85.1 years. This simulates minor measurement or recording variability in continuous clinical data.

### 2.6. Statistical Methods

Reliability and reproducibility were assessed using the intraclass correlation coefficient (ICC). ICC values with 95% confidence intervals were calculated for continuous scores. We applied a two-way mixed-effects model ICC(3,1) to assess reliability, which assumes a fixed set of operators and a single measurement per specimen [20]. This is because the study was planned to quantify reliability under the intended, controlled CLIA operating conditions, including specific CLIA operators and standard operating procedures. ICC(1,1) (one-way random effects) is not appropriate for our crossed design because it does not model operator effects separately. ICC(3,k), which evaluates the reliability of an average of k measurements, is not relevant to clinical practice here because reports are produced from a single operator run. Finally, ICC(2,1) assumes raters are a random sample and estimates generalizability to other raters, which is not the target of this study. Predefined acceptance criteria required an ICC ≥ 0.90 for primary analyses.

Agreement was defined as cases in which ATX risk category was the same after being processed by all operators, in all runs, or in all laboratories. Overall percent agreement with 95% CIs was calculated for binary classifications. Confidence intervals for agreement were computed using Wilson’s method [21]. Predefined acceptance criteria required an agreement ≥ 90% for primary analyses.

All statistical analyses were conducted in Python (v3.10.12). ICCs were calculated using the *pingouin* package [22], and binomial confidence intervals were estimated with *statsmodels* [23]. Cohen’s kappa and corresponding confidence intervals were calculated using the *statsmodels* package [23], while Harrell’s C-index was computed via the concordance statistic implementation in R [24].

## 3. Results

### 3.1. Primary Experiments

In total, 160 cases passed the quality control criteria and had available clinical data. Scanner QC was passed before each run, and no hardware or software issues were identified. The LOB and LOD controls were successfully flagged and no test report was produced for these cases.

Across three runs, intra-operator repeatability assessment of ATX resulted in ICC of 0.99 (95% CI: 0.98–1.00, *p* < 0.01), and agreement of 100% (89–100%). Between two operators, inter-operator reproducibility assessment of ATX resulted in ICC of 0.99 (0.98–1.00, *p* < 0.01), and agreement of 100% (88–100%) and Fleiss’s kappa of 1.00 (1.00–1.00).

All primary repeatability and reproducibility analyses met pre-defined acceptance criteria. Cohen’s kappa also demonstrated perfect agreement of 1.00 (1.00–1.00) for inter-operator agreement.

**Figure 2:**
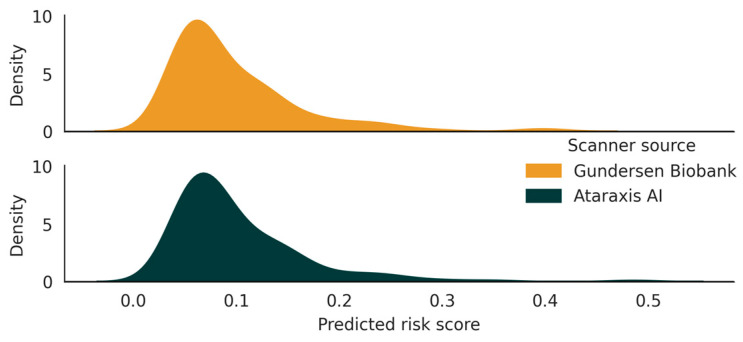
Distribution of calibrated predicted risk scores from two laboratories operating two different scanners. Ataraxis AI CLIA laboratory operated Grundium Ocus 40 and Gundersen Biobank laboratory operated Motic EasyScan Pro 24.

Across two laboratories, ATX had high inter-laboratory (inter-scanner) reproducibility, with an ICC of 0.97 (0.96–0.98, *p* < 0.01), agreement of 94.7% (90.2–97.2%), and Cohen’s kappa of 0.88 (0.80–0.96). The distribution of predicted risk scores is shown in Figure 2, and a summary of the key study results is provided in Table 2.

Given a sample size of 30 subjects, two measurements per subject, and a one-sided significance level of 0.05, the study achieves approximately 95.5% power to detect an ICC of 0.97 relative to a null hypothesis of 0.90. For analyses with three measurements per subject, the corresponding power is approximately 98.8%, confirming that the study is well powered to detect agreement above the pre-defined threshold.

### 3.2. Clinical Validation Bridging Study

In the bridging study, performance of ATX was comparable between the previously reported clinical validation study and analytical validation runs. ATX’s prognostic performance was 0.63 (0.50–0.77) in Ataraxis AI CLIA laboratory and 0.63 (0.50–0.75) in Gundersen Biobank laboratory, compared to the C-index of 0.62 (0.49–0.76) based on the development version of ATX used and previously reported in the clinical validation study.

### 3.3. Clinicopathologic Data Variability

ATX’s prognostic performance remained consistent across 100 resampled datasets with perturbed clinicopathologic data, and achieved an average C-index of 0.62 (0.56–0.68), compared to a C-index of 0.63 (0.50–0.77) when analyzing original data without perturbations. Additionally, ATX had an ICC of 0.85 (0.82–0.88, *p* < 0.01), and an average agreement of 90.0% (85.1–92.4%) between the perturbed and unperturbed predictions. These results demonstrate that the model performance does not deteriorate given realistic perturbation of the data.

## 4. Discussion

This analytical validation demonstrates that Ataraxis Breast RISK produces consistent, reproducible and repeatable risk of recurrence scores across operators and over time, with all metrics meeting predefined success criteria. The QC system reliably identified blank and low-tumor-content specimens, preventing erroneous reporting. The inter-laboratory analysis confirmed that ATX scores are reproducible across independent laboratories using different scanner platforms. The bridging study demonstrated that the CLIA laboratory workflow produces prognostic performance comparable to the prior clinical validation pipeline.

These results are consistent with analytical performance benchmarks reported for established clinical testing modalities [25,26] as well as other AI-based prognostic assays [3,4]. The high ICC values (0.97–0.99) for repeatability and reproducibility indicate that the vast majority of score variance is attributable to true between-patient differences rather than noise introduced in the process of generating input data.

The inter-laboratory ICC of 0.97 is notable given the use of different scanner platforms (Grundium Ocus 40 vs. Motic EasyScan Pro 24). The results indicate substantial agreement, with all discordant cases concentrated near the classification threshold. The pattern is expected and clinically interpretable as cases near the decision boundary inherently have higher classification uncertainty regardless of the analytical platform.

Published studies demonstrate that routine clinicopathologic assessments in breast cancer are highly concordant but not perfectly reproducible. ER, PR, and HER2 status are determined by immunohistochemistry and are subject to variability in tissue fixation and interpretation. For cases near dichotomization cutoff, this can lead to discordance. In the ECOG E2197 study, binary classification-based ER concordance between local and central testing was 90–93%, and PR concordance was 84–90%, indicating measurable misclassification even under controlled conditions [16]. Population-based re-review studies report approximately 6% discordance for ER and HER2 status, and prior HER2 ring studies have observed 5–15% reclassification of locally HER2-positive cases upon central review [17,18]. Interobserver agreement for ductal versus lobular histology is similarly high, approximately 90%, but imperfect [19]. Taken together, these data indicate that real-world discordance rates for key clinicopathologic variables generally fall in the 5–15% range, depending on the biomarker and study context. On this basis, we selected a uniform 10% perturbation probability as a realistic amount of noise to simulate upstream variability.

The clinicopathologic perturbation analysis yielded an ICC of 0.85, which was lower than ICC values in other analyses. However, this analysis quantifies the model’s sensitivity to upstream data quality rather than the precision of the laboratory analytical process itself. The moderate reduction in ICC reflects the expected consequence of introducing 10% error across multiple clinical inputs simultaneously. Additionally, although we attempted to model real-world discordance in hormone receptor status, the perturbation pipeline applied alterations uniformly to every case regardless of baseline expression. In reality, hormone receptor status changes are much more likely to occur in borderline cases and are therefore non-random and conditional on the underlying biomarker level. As a result, our synthetic perturbation likely overestimates the frequency and magnitude of clinically meaningful shifts.

Our study is sufficiently powered to detect an ICC significantly greater than 0.9. However, with only 19 events, it is underpowered to assess prognostic performance of ATX. As a result, the C-indices reported in this manuscript have a wide confidence interval. Importantly, the purpose of reporting the C-indices is not to draw definitive conclusions about prognostic accuracy, but rather to demonstrate that the findings from the original study are numerically reproducible. A more comprehensive validation study would be required to verify that the test generalizes to new patient cohorts.

While our analysis included inter-laboratory validation on two different scanner platforms, this study has not validated the model’s repeatability across all commercially available whole-slide scanners. Future studies should expand the inter-laboratory validation across additional scanner platforms. Additionally, while the clinical validation of the model consists of patients from all major subtypes, the analytical validation study is primarily limited to ER+ HER2- patients. Future analytical validation work should expand to incorporate all other subtypes. Finally, this study relied on retrospectively collected diagnostic slides, so we were unable to prospectively evaluate the effects having multiple specimens or multiple slides derived from the same tissue block. That being said, the retrospective design did provide outcome follow-up that permitted both clinical and analytical validation.

## 5. Conclusions

Ataraxis Breast RISK demonstrated high repeatability, reproducibility, and robust QC behavior in a CLIA laboratory setting, with all predefined acceptance criteria met. Inter-laboratory analysis confirmed cross-platform robustness, and the bridging study demonstrated that CLIA laboratory performance is comparable with the prior clinical validation workflow. The performance of ATX under clinicopathologic data perturbation highlights the importance of clinical data quality while confirming acceptable model robustness. These findings support the analytical readiness of the test for clinical deployment.

## Figures and Tables

**Table 1:** Population statistics of the patients used for bridging study. All values are N (%) unless indicated otherwise. Follow-up time statistics are computed taking into account all patients (censored and not censored).

	Analytical Validation Cohort (N = 160)
Age at diagnosis (Years)	
Median [IQR]	57.5 [50.0–66.0]
Race	
Black or African American	50 (31.25%)
Hispanic or Latino	1 (0.62%)
White	42 (26.25%)
Unknown	67 (41.88%)
ER receptor status	
Negative	1 (0.62%)
Positive	159 (99.38%)
PR receptor status	
Negative	8 (5.00%)
Positive	152 (95.00%)
HER2 receptor status (by immunohistochemistry)	
Equivocal (2+)	1 (0.62%)
Negative (0, 1+)	159 (99.38%)
Pathologic T stage	
T1a	3 (1.88%)
T1b	23 (14.38%)
T1c	68 (42.50%)
T2	51 (31.88%)
T3	4 (2.50%)
T4	1 (0.62%)
Unknown	10 (6.25%)
Pathologic N stage	
N0	108 (67.50%)
N1	36 (22.50%)
N2	2 (1.25%)
N3	2 (1.25%)
Unknown	12 (7.50%)
Recurrence	
No	141 (88.12%)
Yes	19 (11.88%)
Death	
No	157 (98.12%)
Yes	3 (1.88%)
Follow-up time (Years)	
Median [IQR]	4.7 [3.9–6.1]

**Table 2:** Summary of analytical validation results. Across inter-operator, intra-operator and inter-laboratory, the test achieved an agreement greater than 90% and ICC greater than 0.95, passing the pre-specified analytical validation criteria. Additionally, ATX’s performance was robust even when faced with clinicopathologic data variability.

	ICC (with 95% CI)	Agreement (%)
Intra-operator	0.99 (0.98–1.00)	100.0%
Inter-operator	0.99 (0.98–1.00)	100.0%
Inter-laboratory	0.97 (0.96–0.98)	94.7%
Clinicopathologic Data Perturbations	0.85 (0.82–0.88)	90.0%

## Data Availability

The data for this study were generated by Ataraxis AI, Inc. Requests to access the data for reproducing the results reported here can be directed to support@ataraxis.ai.

## Abbreviations

The following abbreviations are used in this manuscript

AI	Artificial Intelligence
ATX	Ataraxis Breast RISK
AV	Analytical validation
CAP	College of American Pathologists
CI	Confidence interval
CLIA	Clinical Laboratory Improvement Amendments
ER	Estrogen receptor
H&E	Hematoxylin and eosin
HER2	Human epidermal growth factor receptor 2
ICC	Intraclass correlation coefficient
IDC	Invasive ductal carcinoma
ILC	Invasive lobular carcinoma
LDT	Laboratory-developed test
LOB	Limit of blank
LOD	Limit of detection
PR	Progesterone receptor
SOP	Standard operating procedure
QC	Quality control
